# Catalytic region mimetics in Na+/H+ exchanger regulatory factor 4 suppress guanylate cyclase 2C activity to regulate enterotoxin triggered diarrhea

**DOI:** 10.1016/j.jbc.2025.110559

**Published:** 2025-08-05

**Authors:** Yashaswini Ramananda, Pramodha Liyanage, Yunjie Huang, Aleksey Porollo, Gerhard Hannig, Anjaparavanda P. Naren, Kavisha Arora

**Affiliations:** 1 Division of Pulmonary and Critical Care Medicine, Department of Medicine, Cedars-Sinai Medical Center, Los Angeles, California, USA; 2 Division of Pulmonary Medicine, Department of Pediatrics, Cincinnati Children's Hospital Medical Center, Cincinnati, Ohio, USA; 3 Department of Pediatrics, Indiana University School of Medicine, Indianapolis, Indiana, USA; 4 Center for Autoimmune Genomics and Etiology (CAGE), Cincinnati Children's Hospital Medical Center, Cincinnati, Ohio, USA; 5 Ironwood Pharmaceuticals, Boston, Massachusetts, USA; 6 Karsh Division of Gastroenterology, Department of Medicine, Cedars-Sinai Medical Center, Los Angeles, California, USA

**Keywords:** guanylate cyclase 2C, cyclic GMP, fluid secretion, secretory diarrheas, sodium-hydrogen exchanger regulatory factor proteins, cystic fibrosis transmembrane conductance regulator, multi-drug resistance protein 4, macromolecular complexes

## Abstract

Guanylate cyclase 2C (GCC) upon binding to the bacterial heat-stable enterotoxin ST, generates excessive cGMP, driving intestinal chloride and fluid secretion that manifests as diarrhea. We investigated the regulatory mechanism of GCC through its interactions with scaffolding proteins sodium-hydrogen exchanger regulatory factor (NHERF)1 to 4. PSD95, Dlg1, ZO-1 (PDZ) domain in NHERF4 inhibited GCC catalytic activity, while NHERF1-3 binary binding had no impact. NHERF4-mediated inhibition was mimicked by two synergistically acting peptides, (N4-110 [NH2-LERPRFCLL-COOH] and N4-195 [NH2-RHAHDVARAQL-COOH]), localized in close proximity within the PDZ1 domain. These peptides, which showed high sequence homology to the GCC catalytic domain, were mapped *via* 3-D structural modeling to the GCC dimer interface. Fluorescence resonance energy transfer (FRET) analysis confirmed that NHERF4-PDZ1 domain binding interfered with GCC oligomerization. In mouse and human enteroid models, NHERF4 peptides dose-dependently reduced GCC-mediated fluid secretion. Additionally, NHERF4-GCC interaction was enhanced upon ST stimulation, suggesting that NHERF4 functions as a negative regulator of aberrant GCC activity during enterotoxin-induced diarrhea. Furthermore, we described a macromolecular complex of GCC with multi-drug resistance protein 4 (MRP4), a cAMP/cGMP efflux transporter, in the regulation of fluid secretion through NHERF3-mediated assembly. Overall, our findings reveal novel regulatory mechanisms for GCC, offering insights into targeted therapies for enterotoxin-triggered diarrheas.

Secretory diarrheas caused by enterotoxigenic *Escherichia coli* (*E. coli*) represent a global health problem that primarily affects young children and a significant number of adults (1, 2). Enterotoxigenic *E. coli* is the leading cause of the most common travel-related illness, termed as Travelers' diarrhea, impacting over three million individuals annually in the United States alone (3, 4). Severe cases of enterotoxin-associated diarrhea often necessitate hospitalization, and post-infectious sequelae, such as irritable bowel syndrome (IBS), have been reported in some patients (5). Pathogenic *E. coli* strain secretes heat-stable enterotoxin ST in the gut, which binds and activates guanylate cyclase 2C (GCC), resulting in elevated levels of intracellular cyclic guanosine-3′-5′ monophosphate (cGMP). Increased intracellular cGMP levels lead to the downstream activation of an ATP-binding cassette (ABC) chloride transporter and channel cystic fibrosis transmembrane conductance regulator (CFTR) through a phosphorylation signaling cascade (6, 7, 8, 9). cGMP also inhibits phosphodiesterase 3, which signals to phosphorylate/inhibit the sodium hydrogen exchanger 3 (NHE3) *via* the cGMP-dependent kinase C type II pathway (10, 11, 12). Upon ST challenge following bacterial infection, excessive chloride secretion through CFTR leads to electrochemically driven paracellular water transport, manifesting as diarrhea (13, 14). GCC can also be physiologically stimulated by endogenous intestinal paracrine hormones, uroguanylin and guanylin (15, 16), which regulate intestinal fluid/ion homeostasis, visceral pain signaling, and intestinal epithelial barrier permeability (17, 18, 19, 20). Consequently, dysregulated GCC activity has been associated with diverse pathological conditions in the gut, such as inflammatory bowel disease (IBD), IBS with constipation, cystic-fibrosis-related constipation, and colorectal cancers (17, 21, 22, 23). Notably, dominant loss (Asp387Gly) and gain-of-function (p.Ser840Ile) mutations in GCC have been linked to meconium ileus (24) and familial diarrhea (25), respectively, highlighting its critical role in gastrointestinal physiology.

Earlier studies suggested that Na^+^/H^+^ exchanger regulatory factor (NHERF) family proteins are involved in both CFTR activation and inhibition of NHE3 in response to ST-induced GCC activation (26, 27, 28). CFTR, a cyclic AMP (cAMP)/cGMP-activated chloride channel expressed in epithelial tissues (29, 30, 31) harbors a carboxyl-terminal PSD95-Drosophila homolog discs–large and tight junction protein ZO-1–binding (PDZ-binding) motif (D-T-R-L_COOH_), enabling CFTR to recognize PDZ domains of multiple adaptor proteins, including the NHERF family of PDZ adaptors (NHERF1-3) (32, 33, 34). These PDZ-dependent interactions of CFTR enable the formation of multi-protein networks involved in a range of physiological and pathophysiological processes, such as the differential effects of NHERF1 (EBP50), NHERF2 (E3KARP), and NHERF3 (PDZK1) on CFTR-related diarrheas and constipation (34).

Multi-drug transporter 4 (MRP4) is another ABC transporter which facilitates the efflux of organic anionic xenobiotic and endogenous molecules such as cAMP, cGMP, eicosanoids, organic anions, bile acids, urate, adenosine diphosphate (ADP), and conjugated steroid hormones (35, 36). MRP4 transporter is integral to the absorption, disposition and excretion of drugs across various tissues, such as the lung, kidney, intestine, ovaries, testis and adrenal glands, where it is primarily expressed in the epithelial cells (35, 36, 37). Notably, MRP4 functionally and physically associates with CFTR, underscoring its significance in the spatial regulation of cAMP signaling and modulation of CFTR-mediated ion secretion (38). Furthermore, MRP4 interacts with CFTR *via* NHERF3 (PDZK1) scaffold protein, forming a macromolecular complex that regulates intestinal fluid secretions, as evidenced by an increased susceptibility to CFTR-mediated secretory diarrhea in MRP4-knockout mice (38). Additionally, MRP4 is functionally associated to intestinal electrolyte and cGMP secretion through GCC/cGMP pathway, activated by linaclotide (39). While MRP4's role in regulating cAMP/cGMP signaling and CFTR activity is well-established, its involvement in modulating GCC mediated cGMP signaling through NHERF proteins and formation of macromolecular complexes remain unclear. Importantly, NHE3 harbors a PDZ binding motif that may facilitate its interaction with MRP4, potentially contributing to the coordinated regulation of fluid homeostasis in the intestinal epithelium.

A previous study reported that NHERF4 (IKEPP/PDZD3) interaction with GCC reduced ST-induced activation of GCC but lacked information on the underlying molecular mechanism for this effect (27). Understanding this mechanism is key to developing strategies to regulate devastating effects of enterotoxin-triggered diarrheas and find specific therapeutic targets. GCC possesses a PDZ-motif (S-T-Y-F_COOH_) in its C-terminal that enables its binding with NHERF proteins (7, 10).

Our data suggested that GCC can engage in PDZ-dependent interactions with the members of the NHERF family (NHERF1-4) with varying tendencies and depending upon the state of GCC activity. The objective of the current study is to investigate macromolecular complexes of GCC that serve as key regulators in the control of enterotoxin-induced diarrheas as well as the physiological activity of GCC.

## Results

### NHERF4 directly inhibits GCC activity

**Figure 1:**
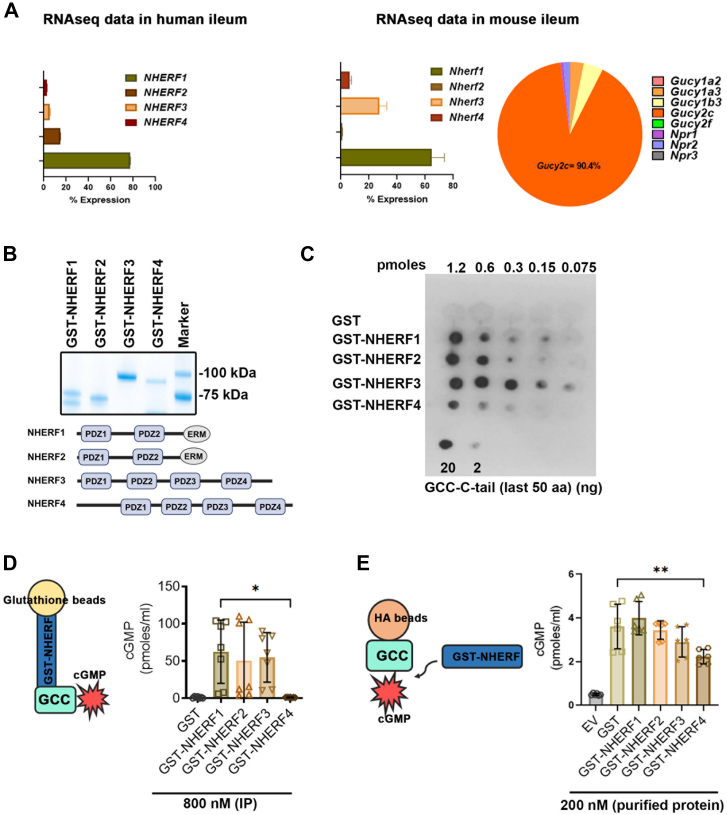
**NHERF4 directly inhibited GCC activity**. *A*, RNAseq analysis showed the relative abundance of *NHERF1* through *NHERF4* in the human small intestine (region-duodenum, whole tissue) on the *left* (three technical replicates from one donor) and the abundance of *Nherf1* through *Nherf4* and Guanylate Cyclase transcripts in the mouse ileum (whole tissue) on the right (three biological replicates). *B*, Coomassie stained gel: purified GST-tagged proteins NHERF1 through NHERF4 from *E. coli.* Below is the illustration of the number of PDZ domains in human NHERF proteins one through four. *C*, dot-blot analysis of GST-NHERF1-4 binding with the C-terminal 50 amino acids of GCC (GCC_C50_) that contains the C-terminal PDZ-domain binding motif STYF_COOH_. GST was used as a negative control. 2 and 20 ng of purified GCC_C50_ were also immobilized on the nitrocellulose membrane as positive controls for detection. The membrane was probed with in-house anti-GCC rabbit polyclonal antibody. *D*, the amount of cGMP was measured using cGMP ELISA with glutathione-conjugated beads that immunoprecipitated GST-tagged proteins previously mixed with the total cell lysate (1 mg protein equivalent) from HEK 293 cells overexpressing GCC. A concentration of 800 nM of the purified NHERF1-4 GST protein was used for immunoprecipitation. GST only protein was used as a negative control. The experiment was performed independently three times, with n = 7 representing each data point. Statistical analysis: one-way ANOVA: interaction (*p* = 0.0015) (F = 5.748). *E*, the amount of cGMP was measured using cGMP specific ELISA in the presence of purified GST proteins (NHERF1-4) used at a concentration of 200 nM and added directly to HA-conjugated beads (10 μl of 10% bead bed volume) with immunoprecipitated HA-tagged GCC from the total cell lysate (1 mg protein equivalent). A 200 nM concentration of the purified protein was incubated with the beads. For control, HA-conjugated beads incubated with lysate from HEK293 cells transfected with pCDNA3 empty vector (EV) were used. The experiment was performed independently three times, with n = 6 representing each data point. Statistical analysis: one-way ANOVA: interaction (*p* < 0.0001) (F = 24.38).

NHERF family of proteins are enriched in the epithelia and control diverse epithelial cell functions including trafficking, transport, and signaling (40). Although there is some overlap in tissue distribution and function among NHERFs, it is well-established that each NHERF protein exhibits distinct properties, translating into unique cellular functions (27, 40, 41, 42, 43, 44, 45). Diversity in the cellular functions of NHERFs may arise as they mediate the organization of distinct high-order signaling complexes (40). We first investigated the relative abundance of NHERFs in the gut as an important determinant of NHERF-mediated regulation of epithelial function. Based on our whole cell transcriptomic data in the human duodenal organoids (hence only epithelial cells), the abundance of NHERF isoforms was found to be in the following order: *NHERF1* (77.11%) > *NHERF2* (14.76%) > *NHERF3* (5.32%) > *NHERF4* (2.79%) (Fig. 1*A*). In contrast, whole cell transcriptomic data from the mouse ileum (separated mucosal epithelium) showed the following order of abundance: *Nherf1* (64.86%) > *Nherf3* (27.57%) > *Nherf4* (6.46%) > *Nherf2* (1.10%) (Fig. 1*A*). Additionally, GCC emerged as the most dominant cGMP-synthesizing enzyme in the gut among membrane as well as soluble GCs (Fig. 1*A*). Given that GCC harbors a putative PDZ-binding motif, we next tested whether GCC interacts with NHERFs that were purified as GST-fusion proteins (Fig. 1*B*). Indeed, GCC interacted with NHERF1 to 4, with the binding order as follows: NHERF3> NHERF1> NHERF2> NHERF4 (Fig. 1*C*). We detected GCC-NHERF (1–4) interaction using immunoprecipitation under saturating binding conditions corresponding to NHERF concentration, *i.e.*, > 1.2 pmoles (Fig. 1*C*). We also developed a method to monitor the activity of GCC coimmunoprecipitated by resin-bound NHERF proteins (Fig 1*D*). We found that GCC immunoprecipitated using GST-NHERF4 exhibited only minimal activity at par with no-GCC in the GST-only immunoprecipitation (Fig. 1*D*). In contrast, GCC immunoprecipitated using NHERF1 through NHERF3 exhibited significantly high activity (Fig. 1*D*). This observation raises the possibility that NHERF4-mediated inhibition may involve either direct or indirect inhibition by another NHERF4 binding protein. To discriminate between these two scenarios, we first immunoprecipitated GCC and incubated it with purified NHERF proteins followed by GCC activity assay on the beads. In fact, externally added purified NHERF4 protein inhibited GCC function relative to GST-only control by 38% (Fig. 1*E*), suggesting that the inhibition of GCC activity is mediated directly by NHERF4. GCC activity in the presence of NHERF1-3 was comparable to the GST-only control. A lower degree of inhibition in this experiment vs. reported in Figure 1*D* could be attributed to the contribution from NHERF4 unbound GCC versus completely NHERF4-bound GCC in Figure 1*D*. In all, these data provide evidence that NHERF4 directly inhibits GCC function, while NHERF1 to 3 exert no apparent effect on GCC activity. Although NHERF4 expression is much lower compared to the other NHERFs within intestinal cells, we anticipate GCC–NHERF4 interaction to be highly compartmentalized and driven by specific physiological cues such as activation of GCC upon enterotoxin challenge.

### NHERF4 inhibits GCC activity through homology-based binding to the catalytic domain

**Figure 2:**
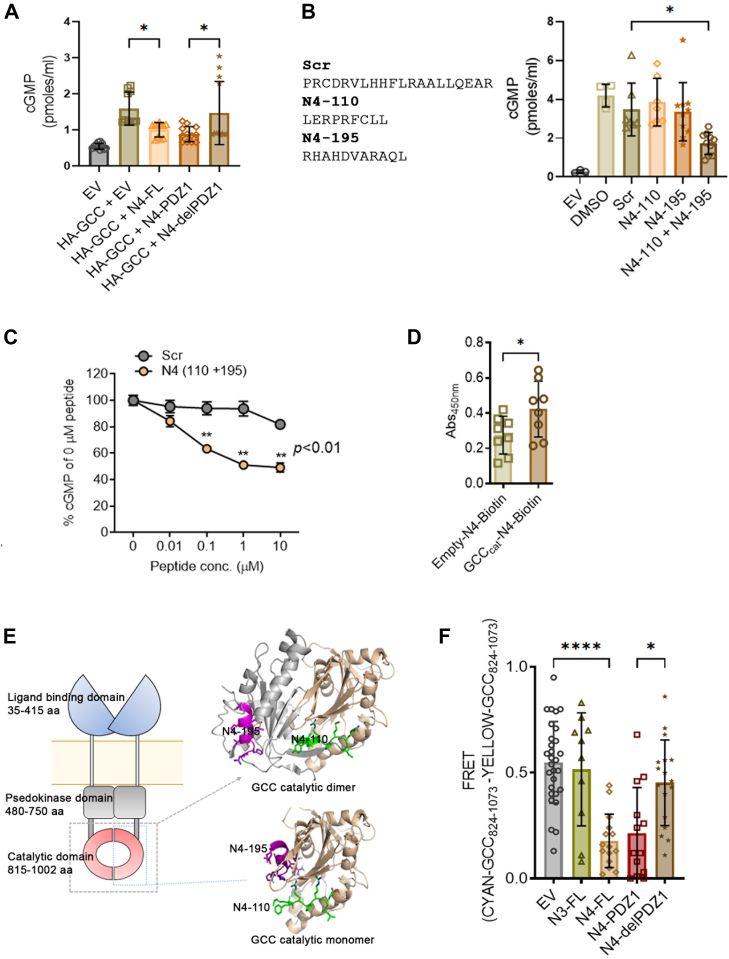
**Catalytic region mimetics in NHERF4 PDZ1 domain inhibited GCC activity and affected CFTR-dependent fluid secretion.***A*, the amount of cGMP was measured using a cGMP specific ELISA with total cell lysate from HEK 293 cells expressing pCDNA3/empty vector (control, EV), HA-GCC + EV (empty vector), HA-GCC + HA-NHERF4 full-length (FL), HA-GCC + HA-NHERF4 PDZ1, and HA-GCC + HA-NERF4 delPDZ1. The experiment was performed independently three times, with n = 10 to 12 representing each data point per condition. Statistical analysis: one-way ANOVA: interaction (*p* < 0.0001) (F = 9.63). *B*, *Left*: NHERF4 peptide sequences N4-110 and N4-195 selected based on their highest homology score with GCC catalytic domain. The scrambled (Scr) peptide was designed with the same amino acid composition as N4-110 and N4-195 combined but with randomized sequence. *Right*: The amount of cGMP was measured using a cGMP specific ELISA on HA-conjugated beads (10 μl of 10% bead bed volume) that immunoprecipitated HA-tagged GCC from the total cell lysate under following conditions: 8 μM Scr, 4 μM N4-110 only, 4 μM N4-195 only, 4 μM N4-110 + 4 μM N4-195. HEK293 cells lysate transfected with pCDNA3 empty vector (control, EV) served as the negative control. The experiment was performed independently three times, with at least two technical replicates per condition. Statistical analysis: one-way ANOVA: interaction (*p* < 0.0001) (F = 8.13). *C*, concentration-dependent effect (0–10 μM) of combination of N4-110 and N4-195 peptides vs. scrambled peptide on the activity of purified GCC *i.e.*, cGMP production. The data were normalized to cGMP concentration at 0 μM peptide/DMSO only. An average mean of n = 8 to 9 data points were represented from three independent experiments. Statistical analysis: *t* test (*p* < 0.01). *D*, HRP-based assay to demonstrate the interaction between GCC catalytic domain (GCC_cat_, amino acids 824–954) and Biotin-N4-195 peptide. GCCcat purified protein (20 μg) was immobilized on the S-beads with 7.5 μM Biotin N4-195 (GCC_cat_-N4-Biotin) subsequently added and allowed to mix overnight. The following day, S-beads were washed extensively with PBS 0.2% Triton-X-100. Protein-peptide interaction was detected using streptavidin HRP. The control condition (Empty-N4-Biotin) consisted of Biotin N4-195 peptide incubated with S-beads in the absence of immobilized GCC_cat_ protein represents each data point n = 8 obtained from three independent experiments. Statistical analysis: *t* test (*p* = 0.0465). *E*, structural architecture of human guanylate cyclase (GCC) and 3D modeling of its catalytic domain. *Left*: A structural diagram of GCC dimer: Extracellular ligand binding domain; pseudokinase domain; guanylate cyclase catalytic domain. Numbers indicated amino acid boundaries of the domains as determined by the NCBI Conserved domain database (CDD) (73). A homodimer of the GCC guanylate cyclase domains (*grey* and *gold*) with GCC regions having sequence homology to inhibitory peptides rendered with side chains as sticks. Highlighted with *green* is the GCC region corresponding to NHERF4 110-LERPRFCLL, *magenta* – to NHERF4 195-RHAHDVARAQ. Predicted binding poses for the NHERF4 inhibitory peptides that hinder homodimerization of GCC. *F*, sensitized FRET assay to measure the effect of various NHERF based constructs (EV, HA-NHERF3, HA-NHERF4, HA-NHERF4 PDZ1, and HA-NHERF4 delPDZ1) on the protein-protein interaction as a function of oligomerization between *cyan*- and *yellow*-tagged GCC catalytic domains used as FRET pair and in the presence of STc (1 μM) stimulation. Quantitation of FRET efficiency from n = 10 to 29, n represents each data point obtained from three independent experiments. Statistical analysis: one-way ANOVA: interaction (*p* < 0.0001) (F = 12.17).

**Figure S1:**
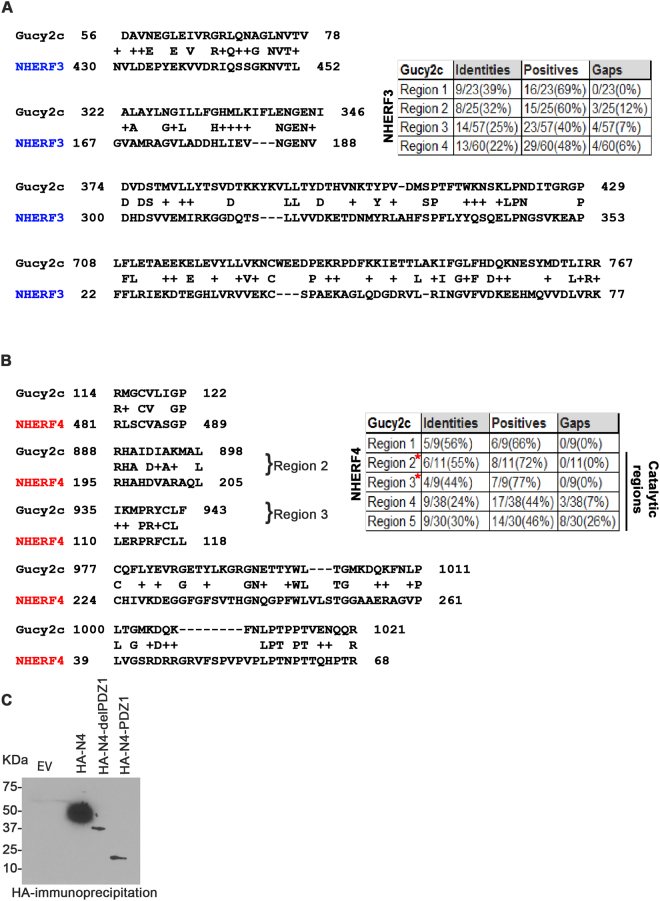
**NHERF4 shares significant sequence homology within GCC catalytic site.***A*, protein BLAST to show the percentage alignment of GCC with the scaffolding proteins NHERF3. NHERF3 did not show any significant sequence similarity with GCC. *B*, protein BLAST to show the percentage alignment of GCC with the scaffolding proteins NHERF4. NHERF4 contains regions especially in PDZ1 domain which exhibit significant similarity to the catalytic regions of GCC. *C*, Western blot data showing the HA-immunoprecipitation of NHERF4 constructs -NHERF4-FL, -PDZ1 and -delPDZ1. Apparant differences in band intensity reflect molar concentration differences, when normalized expression levels were comparable.

Next, we investigated the molecular mechanism underlying NHERF4 mediated inhibition of GCC activity. Based on our earlier finding of direct NHERF4 inhibition, we carefully analyzed the amino acid sequence of NHERF4 and found an unexpected high degree of homology in the PDZ1 domain of NHERF4 to the GCC catalytic domain amino acid sequence. This homology is not present in the other PDZ domains of NHERF4 or in any PDZ domain of NHERF1-3 proteins (Fig. S1, *A* and *B*). Therefore, we considered the possibility of the presence of catalytic mimics in NHERF4-PDZ1 domain that might interfere with the GCC catalytic dimer formation and activity. To begin investigating this possibility, we first generated the following constructs: HA-tagged NHERF4 full-length (FL), NHERF4 PDZ1, and NHERF4 delPDZ1 to test the hypothesis that PDZ1 domain in NHERF4 inhibits GCC activity. Please note that the NHERF4 constructs- NHERF4-FL, -PDZ1, and -delPDZ1 were expressed at comparable levels (Fig. S1*C*). Briefly, recombinant constructs were expressed in HEK 293 cells in the following combinations: HA-GCC + pCDNA3 (empty vector, EV), HA-GCC + HA-NHERF4, HA-GCC + HA-NHERF4 PDZ1, and HA-GCC + HA-NHERF4 delPDZ1 (Fig. 2*A*). Cells were then activated with the GCC agonist STcore (STc) to stimulate cGMP production as described previously (46), and lysed to measure cGMP levels. We observed that HA-NHERF4 co-expression reduced GCC activity (Fig. 2*A*). Co-expression with HA-GCC & NHERF4 PDZ1 reduced GCC activity, and this effect was relieved upon removal of the PDZ1 domain in NHERF4 delPDZ1 when co-expressed (Fig. 2*A*). These data suggests that the PDZ1 domain in NHERF4 is responsible for reducing GCC activity. To further investigate whether the sequences within the NHERF4 PDZ1 domain homologous to those in the GCC catalytic site affect GCC function, we synthesized two peptides in NHERF4 that exhibited the most homology to the GCC catalytic domain sequences: N4-110 [NH2-LERPRFCLL-COOH] and N4-195 [NH2-RHAHDVARAQL-COOH], and the scrambled peptide sequence as NH2-PRCDRVLHHFLRAALLQEAR-COOH containing the same amino acid composition as N4-110 and N4-195 in a scrambled sequence (Figs. S1*B*, 2*B*). Given that NHERF4 PDZ1 suppressed GCC activity, we tested GCC activity in the presence of NHERF4-peptides, N4-110 and N4-195. Addition of either N4-110 or N4-195 peptide to purified GCC did not affect GCC activity; however, the combination of both peptides inhibited GCC function in a concentration-dependent manner (Fig. 2, B and C), suggesting that these two peptides may act synergistically to negatively regulate GCC function. Next, we investigated whether these peptides have the capability to bind to the GCC catalytic domain. We purified GCC catalytic subunit and performed a binding assay with Biotin N4-195 (the most homologous peptide to GCC catalytic sequence). No biotin N4-195 was used as a negative control. Indeed, we detected GCC-N4-195 binding based on a significant HRP-binding signal relative to the control (Fig. 2*D*). Here, we concluded that GCC catalytic mimics in NHERF4- PDZ1 domain inhibit GCC activity based on a direct protein-protein interaction. Also, it alludes to the possibility that NHERF4 interacts with GCC at multiple sites of the GCC protein, *i.e.*, C-terminal motif as well core catalytic sites.

### GCC oligomerization as required for catalytic function is perturbed by NHERF4

As GCC oligomerization is essential for the catalytic activity of GCC (47), we hypothesized that the NHERF4 peptides inhibit GCC function by preventing GCC oligomerization. We first investigated this possibility by performing protein-peptide docking studies. A 3D model of the guanylate cyclase domain of human GCC was built using a homology-based modeling of eukaryotic catalytic domain of guanylate cyclase as a template (PDB ID: 3ET6) (Fig. 2*E*). ClusPro was used for protein-protein docking (48). All subsequent protein structure analysis and visualization were conducted using PyMol (http://www.pymol.org/). From the UniProt domain annotation of human NHERF4, the experimentally identified inhibitory peptides 110-LERPRFCLL and 195-RHAHDVARAQ are located at the –NH2 and –COOH termini of the PDZ one domain, respectively (UniProt ID: Q86UT5/Q86UT5-2). From a wide variety of published 3D structures of PDZ domains (Pfam and PDB databases), these termini are in close spatial proximity to each other in the domain, which may suggest their simultaneous involvement in the interaction with GCC in the native structure of NHERF4 (Fig. 2*E*). When GCC regions corresponding to inhibitory NHERF4 peptides are mapped into the dimeric structure of the catalytic domain, GCC-to-N4-110 appears to be at the protein–protein interaction interface of the dimer, whereas GCC-to-N4-195 is located on the opposite site of the structure (Fig. 2*E*). Hence, the inhibitory role of N4-110 can be explained by disrupting a dimerization of GCC catalytic domains through a competitive binding to each of the subunits of the GCC catalytic domain. On the other hand, localization of the GCC-to-N4-195 region cannot be attributed to binding to the dimerization interface. A Protein Databank-wide search also did not reveal any other protein-protein interactions at this site that could be affected by N4-195. Moreover, this GCC site is distant from the GCC-to-N4-110 region, so it is unlikely that the stable N4 PDZ1 domain unfolds to accommodate such remote spatial locations for its termini. To predict possible binding sites for N4-195, a protein-protein docking was performed between human GCC monomeric domain and this peptide. According to ClusPro, the top-scoring binding mode for N4-195 (both in terms of estimated binding energy and the occupancy of the cluster) is located next to the predicted binding site of the N4-110 peptide at the homodimeric interface of GCC (Fig. 2*E*). This is a scientifically feasible explanation of how the peptide mimics would synergistically inhibit GCC activity *i.e.*, by binding GCC catalytic site in monomeric state and interfering with the dimer formation upon STc binding. To test the hypothesis that NHERF4 interferes in the formation of GCC oligomer, we sorted to fluorescence resonance energy transfer (FRET) assay to measure the effect of NHERF4 constructs on the interaction between GCC catalytic units as FRET pair. Cyan- and yellow-tagged GCC catalytic domains were co-expressed with pCDNA3 (empty vector, EV), HA-NHERF3, HA-NHERF4, HA-NHERF4 PDZ1, and HA-NHERF4 delPDZ1 and sensitized FRET was performed as a measure of protein-protein interaction between Cyan-GCC_cat_ and Yellow-GCC_cat_ in the presence of STc stimulation. We observed that co-expression of NHERF4 dramatically reduced FRET efficiency of GCC_cat_ oligomerization (0.17 ± 0.03) vs. control (0.54 ± 0.03), while NHERF3 had no effect (0.51 ± 0.08) (Fig. 2*F*). In a similar manner to NHERF4 FL, NHERF4 PDZ1 inhibited FRET efficiency of GCC_cat_ oligomerization (0.21 ± 0.05) while this effect recovered upon removal of PDZ1 domain in NHERF4 delPDZ1 (0.45 ± 0.04) (Fig. 2*F*). The FRET-based GCC_cat_ oligomerization data coincides with the effect of NHERF4 constructs on GCC activity as shown in Figure 2*A*. Overall, we determined that NHERF4 perturbs GCC oligomerization that is required for GCC enzymatic activity and propose this as a likely mechanism by which NHERF4 suppresses GCC function.

### NHERF4 reduces ST-induced fluid secretion in the enteroid model

To test the potential implications of NHER4-mediated inhibition of GCC function in enterotoxin-induced diarrhea, we used enteroid model to test the effect of NHERF4 on ST-induced CFTR-dependent fluid secretion. We have developed physiologically relevant enteroids/colonoids/intestinal organoids models from the crypts of the small intestine, colon, and rectal tissues to study CFTR-dependent fluid secretion (49, 50, 51, 52). The advantages of organoid models include **(i)** they are simple and miniature 3D version of an organ containing all key epithelial sub-types; **(ii)** addition of cAMP agonists such as Forskolin (FSK, 2–10 μM) as well as cGMP agonists (STc) results in highly robust fluid secretion response marked by organoid swelling that is largely CFTR-dependent (46, 51, 53, 54). While the organoid model provides a convenient measure of CFTR function through fluid secretion physiologically associated with diarrhea, it is an indirect method and cannot directly measure electrogenic activity, as done in the gold-standard assay for CFTR through Ussing chambers (55, 56).

**Figure 3:**
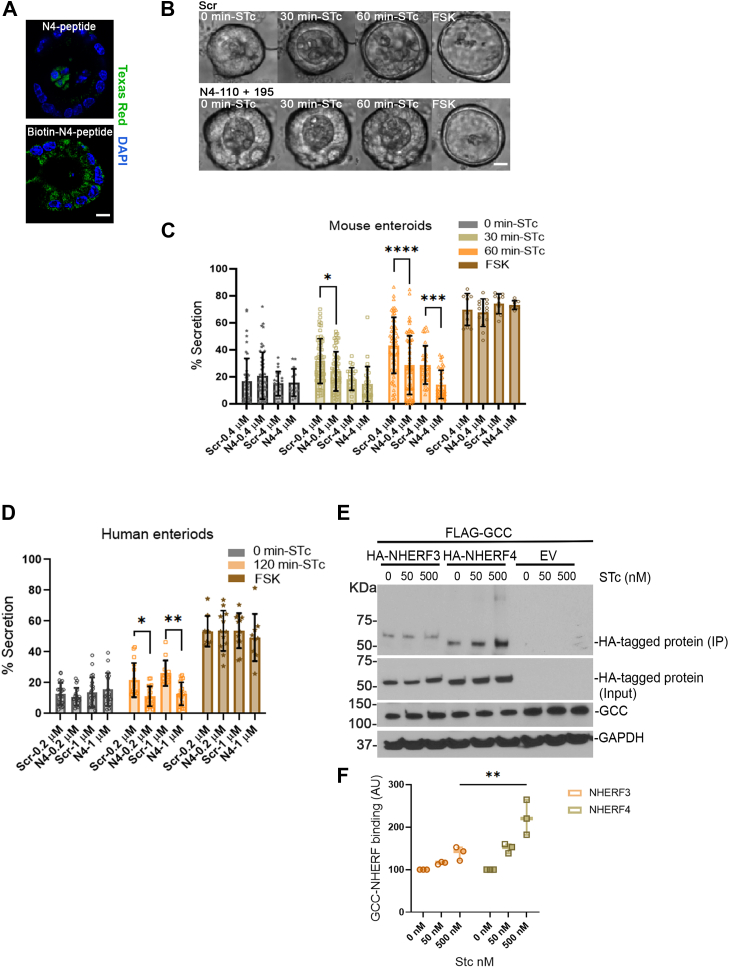
**Catalytic region mimetics in NHERF4 affected CFTR-dependent fluid secretion in the mouse and human enteroid model**. *A*, Day 1 intestinal mouse enteroids were treated with Chariot- N4-195 or Chariot-Biotin-N4-195 peptide mix, fixed and incubated with Streptavidin conjugated Texas red (pseudocolor-*green*). Confocal images were obtained depicting successful delivery of the peptide into the enteroids. Variability in the concentrations of the delivered peptide inside the enteroids was noted (Scale bar: 5 μm). *B*, representative bright field images of Day 1 mouse intestinal enteroids subjected to Chariot peptide uptake assay, using Scr peptide or NHERF4 peptide combination N4-110 + N4-195 (N4) at various doses. After 2 h of incubation with the peptides-chariot mix, enteroids were subjected to fluid secretion assay in which CFTR dependent secretion was stimulated with STc (500 nM) for up to 60 min. N4 peptide combination was observed to perturb STc/cGMP stimulated CFTR dependent secretion without perturbing FSK/cAMP (10 μM) stimulated secretion (Scale bar: 10 μm). *C*, *Bar-graph* represents the quantitation of STc (1 μM) and FSK (10 μM) stimulated fluid secretion in the mouse enteroids which were subjected to Scrambled peptide (Scr) and NHERF4 peptide combination N4-110 + N4-195 (N4) at two concentrations (0.4 μM & 4 μM). Fluid secretion in the mouse enteroids calculated from n = 15 to 60 organoids per condition obtained from three mice. Statistical analysis: two-way ANOVA: interaction (*p* < 0.0001) (F = 4.585). *D*, *Bar-graph* represents the quantitation of STc(1 μM) and FSK(10 μM) stimulated fluid secretion responses in human duodenal enteroids which were subjected to Scrambled peptide (Scr) and NHERF4 peptide combination N4-110 + N4-195 (N4) at two concentrations (0.2 μM & 1 μM). Fluid secretion in the human enteroids calculated from n = 10 to 26 organoids per condition obtained from three technical replicates from a single donor. Statistical analysis: two-way ANOVA: interaction (*p* = 0.0055) (F = 3.177). *E*, HEK-293 cells that overexpressed FLAG-GCC in combination with empty vector (EV), HA-NHERF3 and HA-NHERF4 were subjected to STc (0, 50 and 500 nM) for 30 min. FLAG-GCC was immunoprecipitated and interaction of GCC with the NHERF proteins was detected using anti-HA antibody. GAPDH was used as a loading control. NHERF4 interaction with GCC correlated with the level of stimulation of GCC. *F*, *Box plot* shows binding of NHERF proteins with GCC across the increasing concentrations of STc (0 nM, 50 nM & 500 nM) to capture different activation states of GCC. Data obtained from three independent experiments. The mean *gray* value for each band was calculated using ImageJ. Statistical analysis: two-way ANOVA: interaction (*p* = 0.0076) (F = 7.53).

To study the effect of N4 peptides that were earlier shown to inhibit GCC activity, on CFTR-dependent fluid secretion, we chose the chariot peptide delivery method to deliver these peptides into the mouse and human enteroids (Fig. 3*A*). Chariot is a delivery reagent that efficiently transports biologically active proteins, peptides, and antibodies directly into cells (57, 58). Fluid secretion was monitored in murine and human enteroids in response to STc and FSK following delivery of N4-scrambled or N4-110 plus N4-195 peptides using chariot delivery. We observed that N4-peptides significantly reduced STc fluid secretion in a concentration-dependent manner in both human and murine enteroids, while FSK-stimulated secretion remained unaffected in the N4-peptide-treated enteroids, indicating that total CFTR activity remains unaffected through the cAMP pathway (Fig. 3, *B*–*D*). N4-scrambled peptides did not reduce STc-stimulated secretion at lower concentrations, while some non-specific inhibition was observed at high concentrations (Fig. 3*C*). However, human enteroids exhibited a slower rate of swelling, albeit a statistically significant change compared to the murine organoids. Based on this observation, we concluded that the N4 peptides inhibited fluid secretion upon STc challenge through the cGMP pathway of activation for CFTR.

We next investigated whether the activation of GCC alters the binding to NHERF3 and NHERF4 that may have implications to how the inhibitory effect of NHERF4 on GCC activity is relevant to enterotoxin-triggered diarrheas. Interestingly, with an increasing concentrations of STc, there was an enhanced binding of GCC with NHERF4, while the binding with NHERF3 was not affected under these conditions (Fig. 3, *E* and *F*). Therefore, we concluded that enhanced binding of NHERF4 to GCC upon enterotoxin challenge and GCC activation could be a key inhibitory mechanism to regulate excessive fluid transport.

### GCC and MRP4 are functionally coupled in the intestinal epithelial cells

**Figure 4:**
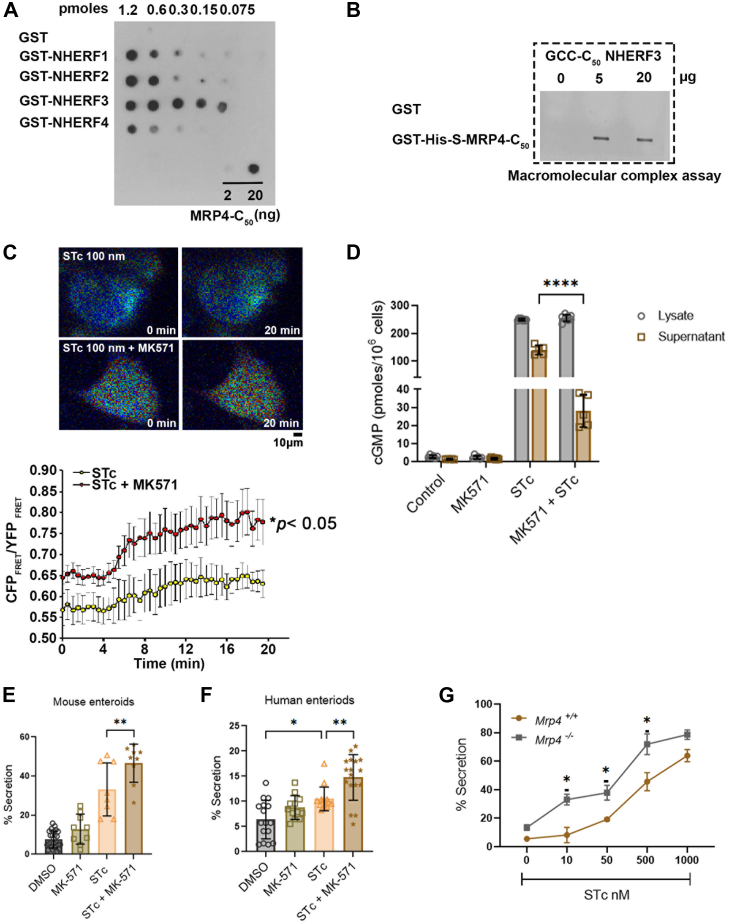
**GCC and MRP4 are functionally coupled in the intestinal epithelial cells**. *A*, dot-blot analysis of GST-NHERF1-4 binding with the C-terminal 50 amino acids of MRP4 (MRP4_C50_) that contains the C-terminal PDZ-domain binding motif ETAL_COOH_. GST was used as a negative control. 2 and 20 ng of purified MRP4_C50_ were also immobilized on the nitrocellulose membrane as positive controls for detection. The detection of the protein interaction was performed using S-protein HRP Conjugate. *B*, the macromolecular complex formation assay between MRP4_C50_ and GCC_C50_*via* NHERF3. *C*, *Top*: Representative pseudocolor images to mark production of cGMP in the presence of STc (100 nm) or Stc (100 nm) + MK571 (10 μM) in T-84 cells using ratiometric FRET sensor Cygnet 2.1. *Bottom*: *Line graph* depicts time-lapse measurement of ratiometric FRET T-84 cells treated with STc (100 nm) or Stc (100 nm) + MK571 (10 μM). Statistical analysis: *t* test (*p* = 0.0481). *D*, *bar-graph* represents the amount of cGMP measured using ELISA from total cell lysate and the supernatant (medium) from T-84 cells treated with ± STc (1 μM, 30 min) ± MK571 (10 μM, 30 min). n represents each data point with n = 6 to 8 obtained from three independent experiments. Statistical analysis: two-way ANOVA: interaction (*p* < 0.0001) (F = 584.5). *E*, *bar-graph* represents the quantitation of fluid secretion in the mouse enteroids treated with ± STc (1 μM, 30 min) ± MK571 (10 μM, 30 min). Fluid secretion in the mouse enteroids calculated from n = 8 to 25 organoids per condition. Statistical analysis: one-way ANOVA: interaction (*p* < 0.0001) (F = 63.96). *F, bar-graph* represents the quantitation of fluid secretion in the human enteroids treated with ± STc (1 μM, 30 min) ± MK571 (10 μM, 30 min). Fluid secretion in the human enteroids calculated from n = 12 to 17 organoids per condition. Statistical analysis: one-way ANOVA: interaction (*p* < 0.0001) (F = 16.44). *G*, *line-graph* represents the quantitation of fluid secretion in *Mrp4*^*+/+*^ and *Mrp4*^*−/−*^ mouse enteroids treated with STc (1 μM, 30 min). Fluid secretion in the mouse enteroids calculated from n = 7 to 20 organoids per condition. Statistical analysis: two-way ANOVA: interaction (*p* < 0.008) (F = 1.97).

Although NHERF3 binding to GCC did not affect GCC activity in enterotoxin-induced diarrhea, NHERF3 has been demonstrated to bind to MRP4, a cGMP/cAMP efflux transporter, with high affinity relative to other NHERF proteins (Fig. 4*A*). This interaction is relevant to diarrhea as it negatively regulates CFTR function in the gut (38). MRP4 interacted with NHERF1 to 4, with the binding order as follows: NHERF3> NHERF2≥ NHERF1> NHERF4 (Fig. 4*A*). Our data suggested that GCC could exist in a macromolecular complex with MRP4 *via* NHERF3 (Fig. 4*B*). Briefly, in this assay, the last C-terminal 50 amino acids of GCC and MRP4 containing intact PDZ motifs were used to detect complex formation using purified NHERF3 (Fig. 4*B*). This data alludes to the possibility that GCC-NHERF3-MRP4 are functionally coupled and could negatively regulate Travelers' diarrhea *via* a distinct mechanism than NHERF4. Indeed, based on ratiometric FRET to monitor live cGMP production using a cGMP-FRET sensor cygnet 2.1 (59), we observed a synergistic accumulation of cGMP in the cells expressing GCC and treated with MRP4 inhibitor MK571 (38, 60) (Fig. 4*C*). We additionally observed reduced cGMP concentrations (in the cell supernatant) in the presence of MK571 in GCC overexpressing cells stimulated with STc suggesting reduced cGMP efflux through MRP4 (Fig. 4*D*). Treatment of both mouse and human enteroids with MK571 led to enhanced STc-stimulated fluid secretion (Fig. 4, *E* and *F*). We additionally monitored dose-dependent STc-induced secretion in *Mrp4* KO and WT mouse enteroids and observed increased fluid accumulation in *Mrp4* KO stimulated with STc <1000 nM (Fig. 4*G*). These data suggested that GCC and MRP4 are functionally coupled and regulate fluid secretion in intestinal cells. These findings demonstrate the presence and significance of highly specialized macromolecular complexes in controlling diarrheal diseases.

## Discussion

**Figure 5:**
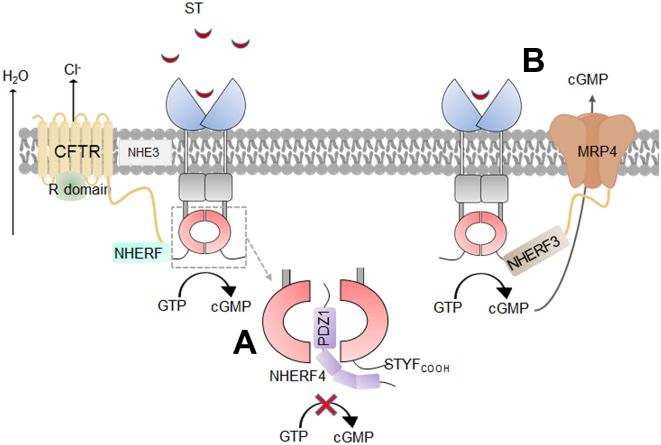
**Model of the study.** GCC catalytic mimics in NHERF4 PDZ1 domain prevent GCC dimerization by becoming incorporated into the GCC catalytic dimer pocket, while the primary recognition occurs through C-terminal PDZ motif in GCC (mechanism A). This interaction is of major significance under enterotoxin challenge to prevent excessive fluid loss through CFTR-coupled chloride secretion. In another scenario, a macromolecular complex among GCC-NHERF3-MRP4 regulates GCC activity under normal physiologic conditions by regulating the export of cGMP through MRP4 (mechanism B). Inhibition of MRP4 *via* MK571 stimulates CFTR-dependent fluid secretion through compartmentalized accumulation of cGMP.

Enterotoxin-triggered secretory diarrheas are a significant cause of morbidity and mortality in children in the developing world and a common travel related illness. Here, we report for the first time that GCC catalytic mimetic sequences in NHERF4 are involved in the negative regulation of GCC enzyme activity, a novel mechanism of functional regulation of GCC that bears relevance to enterotoxin-triggered diarrheas. The adoption of synthetic strategies such as biological protein mimetics and foldamers to inhibit or enhance the activity of the enzyme has been reported (61), alluding to a point of sequence or conformation specific biological substitutes capable of functional modulation of an enzyme. To the best of our knowledge, a natural occurring mechanism such as the NHERF4-GCC interaction has not been reported previously. In this study, we have demonstrated that NHERF4-mediated inhibition of GCC activity could also be mimicked by two synergistically acting peptides in NHERF4 with high sequence homology to the GCC catalytic sites. Additionally, enhanced binding of NHERF4 to active GCC oligomer suggests that NHERF4 acts as a physiological and context-dependent negative regulator of GCC activation (Fig. 5). This mechanism is important to prevent devastating loss of fluid and salt from the body upon enterotoxin challenge.

Protein scaffolds, such as NHERF family proteins, enable the formation of multi-protein complexes and are crucial for short-term and dynamic regulation (62). Our data suggested that GCC-NHERF4 complex formation occurs in response to excessive GCC activation, a mechanism relevant to enterotoxin-induced diarrhea. NHERF4 has been shown to activate NHE3, contributing to enterotoxin-driven diarrhea (63). Therefore, multiple NHERF4 complexes might act together to effectively mitigate enterotoxin-induced diarrhea. ST-mediated inhibition of Na/H exchanger (NHE3)-GCC-cGMP signaling is also regulated *via* NHERF2-NHERF3 heterodimerization (64). Although we have not yet investigated the role of NHERF heterodimers in regulating GCC function, we believe that these heteromeric interactions, potentially involving a NHERF3-NHERF4 heterodimer, could be crucial for the assembly of multiple higher-order complexes, including CFTR, MRP4, and NHE3s that are necessary for the acute and dynamic regulation of GCC's physiological and pathophysiological responses. The current data, combined with earlier findings from our laboratory, underscore the relevance of macromolecular complexes in controlling diarrheal diseases. Moreover, the homeostatic regulation of this macromolecular complex could be highly relevant not only in diarrheal diseases but also in chronic and age-related constipation as GCC is a target of therapy for constipation through Linaclotide© (STc) (65, 66, 67, 68).

Our data indicated that NHERF3 had the highest binding affinity for GCC among the NHERF proteins. However, under stimulatory conditions, GCC preferentially bound to NHERF4. Li *et al.* demonstrated that NHERF3/PDZK1/CAP70 binds to MRP4, a key cAMP/cGMP efflux transporter in the gut (38). Our study revealed a novel regulatory mechanism involving the formation of multiple macromolecular complexes among GCC, NHERF3, and MRP4, which together play a pivotal role in controlling fluid secretion in the gut (Fig. 5). The increased fluid secretion observed in *Mrp4*-deficient enteroids highlights the inhibitory role of MRP4, further supporting its importance in regulating intestinal homeostasis.

In this study, we used two peptides, N4-110 and N4-195, derived from the NHERF4 PDZ1 domain sequence, which exhibited the highest degree of homology to specific sequences in the GCC catalytic domain. We observed a synergistic inhibitory effect on GCC activity using these peptides. Endogenous delivery of these peptides for a therapeutic pursuit for enterotoxin-induced diarrhea could be challenging. A medicinal chemistry strategy of synthesizing “peptide mimetics” or “peptidomimetics” (69) that involves the generation of molecules such as a modified peptide with a majority of a synthetic backbone to facilitate their delivery in biological systems could evolve as an important pharmacological approach to regulate GCC macromolecular complex formation in enterotoxin-induced diarrhea. We believe that targeting GCC macromolecular complexes is attractive, given the multiple functions of GCC in the gut and its involvement as a key receptor to mediate enterotoxin-induced diarrheas. Based on this knowledge, the therapeutic value of this approach is not only limited to the management of secretory diarrheas but also to other gastrointestinal diseases, including constipation or diarrhea-predominant IBS and IBDs.

## Experimental procedures

### Mice

Wild-type mice (6–12-week-old) used in the study were either from in-house colonies or purchased from Jackson Laboratories (Bar Harbor, ME). *Mrp4* KO mice (6–12-week-old) were originally generated by Dr John D. Scheutz laboratory at St Jude, Memphis, TN (70). The mice were maintained in a barrier facility at Cincinnati Children's Hospital Medical Center and were fed normal chow.

### Statistics

The statistical significance was assessed using an unpaired *t* test, one-way ANOVA, or two-way ANOVA, as appropriate. For experiments involving multiple group comparisons, Turkey's *post hoc* test was applied for *p*-value adjustment. A *p*-value <0.05 was considered statistically significant. All the experiments were independently repeated at least three times, and details of each data point are provided in the corresponding figure legends. All the data are presented as mean ± SD, unless otherwise stated. The levels of significance are denoted as follows:

∗*p* < 0.05.

∗∗*p* = 0.001.

∗∗∗*p* = 0.0001.

∗∗∗∗*p* < 0.0001.

### Chemicals and antibodies

Antibodies in this study included anti-CFTR antibodies R1104 [Eric Sorscher lab, CF Center, University of Alabama, Birmingham, AL (presently, Emory University)], 24-1 (Bio-Techne), anti-HA (#3724 Cell Signaling Technologies, Danvers, MA), and anti-FLAG HRP (A8592, clone M2, Sigma, St Louis, MO). STc was custom-synthesized by American Peptide Company.

CFTRinh172 was purchased from Sigma. MK571 was obtained from Cayman Chemical.

GST-His-S-fusion proteins for C-terminal 50 a.a. of MRP4 (GST-His-S-MRP4-C50) and GCC were generated using pET41 vector (Novagen), and His-S-fusion protein of full-length PDZK1 was generated using pET30 vector (Novagen).

### Purification of GST-NHERF1, 2, 3, and 4

GST-fusion protein for full-length PDZ proteins (NHERF1 to 4) was generated using the pGEX vector according to the manufacturer's instructions (Amersham Pharmacia). GST-tagged NHERF1, NHERF2, NHERF3, and NHERF4 constructs were transformed into *E. coli* BL21 and Origami competent cells, respectively. Bacterial cultures were grown, and protein expression was induced by the addition of 0.2 mM IPTG for 4 h at 37 °C. Following induction, cells were harvested by centrifugation and resuspended in lysis buffer containing 50 mM Tris (pH 7.2), 1 mM EDTA, 10% sucrose, and 1 mg/ml lysozyme. The mixture was incubated on ice for 30 min to allow for cell lysis. To solubilize membrane-bound proteins, 2.1% Triton X-100 was added, and the lysate was further incubated on ice for an additional 30 min.

The lysed cells were then centrifuged, and the resulting supernatant was incubated with 100 μl of Glutathione Sepharose 4B beads (Thermo Scientific) for 2 to 3 h at 4 °C with gentle rotation. The beads were washed extensively in wash buffer containing protease inhibitors, and the GST-NHERF fusion proteins were eluted using 20 mM glutathione. Protein concentrations were determined by measuring absorbance at 280 nm.

### Cell transfection

HEK 293 cells were obtained from ATCC and were prepared in regular medium (DMEM/F-12 10% FBS, 1% penicillin-streptomycin). Human GCC cDNA was a kind gift from Dr. Kris A. Steinbrecher, Cincinnati Children's Hospital Medical Center. Cells were transfected with human HA-GCC (HA tag was inserted at amino-acid position 39) cDNA by using Lipofectamine 3000 (Thermo Fisher Scientific) according to the manufacturer's protocol. The transfected cells were studied after 48 h.

T-84 cells were obtained from ATCC and cultured in DMEM/F-12 10% FBS, 1% penicillin-streptomycin. Cells were transfected with the ratiometric FRET senor Cygnet 2.1 (59) using Lipofectamine 3000 (Thermo Fisher Scientific) according to the manufacturer's protocol.

### Cell lysate preparation and GCC immunoprecipitation

HEK 293 cells stably expressing HA-GCC were lysed in PBS 0.2% Triton X-100 (for membrane protein solubilization) containing protease-inhibitor cocktail (1 μM aprotonin, 1 μM leupeptin, and 1 mM phenylmethylsulfonyl fluoride). HA-GCC was immunoprecipitated from whole-cell lysates by using HA-conjugated resin (Sigma). Proteins immobilized on beads were eluted using a low-pH, glycine-based elution buffer containing 0.2% Triton-X-100. Samples were incubated for 10 min at 37 °C and subjected to SDS–PAGE and Western blot following standard protocols.

### Intestinal crypt isolation and quantitation of fluid secretion in enteroids

Preparation of the mouse intestinal crypt and quantitation have been thoroughly described previously (71). Human crypts were isolated and cultured as reported earlier (72).

### Measurement of whole-cell cGMP and cGMP formation by immunopurified GCC

HEK 293 cells expressing HA-GCC or without the expression of GCC, grown in a 60 mm dish, were washed three times with pre-warmed PBS containing calcium and magnesium and treated with IBMX for 10 min before stimulation with STc 500 nM for 30 min at 37 °C. To calculate intracellular cGMP, cells were lysed in 0.1 N HCl 0.2% Triton-X-100 and centrifuged at 800*g*, and the supernatant was collected and used for cGMP-specific ELISA following the manufacturer's protocol (Enzo Life Science, Farmingdale, NY) (catalog number: 89141-068).

Briefly, samples and standards were added to the wells, followed by incubation with blue cGMP conjugate. After incubation with the supplier provided cGMP antibody, the wells were washed three times with the supplied wash buffer. pNpp substrate was added, and the plate was incubated for 1 h. Finally, the reaction was stopped, absorbance was measured at 405 nm. cGMP concentrations were determined by plotting sample optical density against the standard curve.

To measure extracellular cGMP, cells were washed three times with pre-warmed PBS containing calcium and magnesium and replaced with 1 ml PBS. Cells were treated with IBMX for 10 min before stimulation with STc (500 nM) ± MK571 (10 μM) for 30 min at 37 °C. Supernatant was collected after 30 min to measure extracellular cGMP, and cells were placed on ice to be later lysed for measuring intracellular cGMP as described above.

To measure cGMP formation by immunopurified GCC, HA-GCC was immunoprecipitated on HA beads from the whole cell lysate from HEK 293 cells grown on four to six 100 mm culture dishes, and overexpressing HA-GCC. 20 μl aliquots of HA-GCC bead resin were prepared per sample. The following reaction mixture was added to the beads to initiate the reaction: 100 μM GTP, 100 μM IBMX, and 1 μM STc in 120 μl PBS containing calcium and magnesium. The beads were incubated at 37 °C for 30 min. The reaction was stopped by placing the beads immediately on ice. To measure cGMP, the beads were spun down at 6000 rpm in a cooled centrifuge, and 100 μl of the sample was carefully collected. cGMP was measured using ELISA as mentioned before.

### Normalized and ratiometric FRET

FRET studies were performed as described previously (50). Briefly, HEK 293 cells grown at 60% confluence were transfected with Cyan- and yellow-tagged GCC catalytic domains with co-expression of pCDNA3 (empty vector), HA-NHERF3, HA-NHERF4, HA-NHERF4 PDZ1, and HA-NHERF4 delPDZ1. Sensitized FRET was performed as a measure of protein-protein interaction between Cyan-GCC_cat_ and Yellow-GCC_cat_. To perform ratiometric FRET, cGMP sensor cygnet 2.1 was expressed in HEK293 cells (59).

### Delivery of NHERF4 or Scr peptides and immunostaining

Peptide delivery (NHERF4 or Scr peptides) was performed using the Chariot system (Active Motif), according to the manufacturer's instructions (57, 58). Briefly, 4 μM of the peptide was mixed with the Chariot solution in a total volume of 400 μl and incubated at room temperature for 30 min. The Chariot-peptide complex was then added to the media containing mouse and human enteroids and incubated for 2 h at 37 °C in a humidified atmosphere with 5% CO_2_. Following the incubation, enteroids were subjected to a fluid secretion assay without removing the media, in which CFTR-dependent secretion was stimulated using STc (500 nM) for up to 60 min.

For immunostaining, mouse enteroids treated with either Chariot-N4-195 or Chariot-Biotin-N4-195 peptides were fixed and incubated with Streptavidin-conjugated Texas-Red. The efficiency of peptide delivery was assessed using confocal microscopy (Olympus FV1200), which confirmed successful peptide uptake into the enteroids.

### RNA Extraction for Bulk-RNA sequencing

RNA from mouse (ileum) and human (duodenum) small intestine tissues was isolated using RNeasy Isolation Kit (Invitrogen, Cat#74004) according to the manufacturer's instructions. Samples were sequenced by the Genomics, Epigenomics, and Sequencing Core at the University of Cincinnati. The sequenced dataset was analyzed using the BaseSpace Sequence Hub app. First, RNA-Seq Alignment (v2.0.2) was performed, followed by RNA-Seq Differential Expression analysis (v1.0.1). STAR was used for alignment, and Salmon was employed for quantification of gene expression (Transcripts Per Million, TPM).

### Macromolecular complex assay

A Slot blot-based experiment was performed to assess the formation of a macromolecular complex involving MRP4, NHERF3, and GCC. GST-His-S-MRP4-C-terminal 50 aa fusion protein (0, 5 & 20 μg) and GST alone (negative control) were applied to nitrocellulose membrane and allowed to air-dry. The membrane was then washed once with TBST (TBS + 0.1% Tween-20), blocked with 5% non-fat dry milk in TBST, and incubated overnight at 4 °C with 5 μg of GST-NHERF3 under constant mixing. The following day, after thorough washing, the membrane was further incubated with 0.5 μg purified Flag-GCC-C-terminal 50 aa at 4 °C with gentle agitation. Following extensive washing, the macromolecular complex was detected using a GCC-C-terminal specific antibody.

### HRP- based binding assay

To demonstrate the interaction between GCC catalytic domain (GCC_cat_, amino acids 824–954) and Biotin-N4-195 peptide, 20 μg GCCcat purified protein was immobilized on the S-beads and incubated overnight at 4 °C with 7.5 μM Biotin N4-195 (GCC_cat_-N4-Biotin) under gentle agitation. The following day, S-beads were washed extensively with PBS 0.2% Triton-X-100 to remove unbound peptide. Protein-peptide interaction was detected using streptavidin HRP followed by incubation with TMB substrate solution, and absorbance was measured at 450 nm. The control condition (Empty-N4-Biotin) consisted of the Biotin N4-195 peptide incubated with S-beads in the absence of immobilized GCC_cat_.

## Study approval

Human studies: Patient samples for research were obtained under an approved protocol from the Cedars-Sinai Medical Center (CSHS) Institutional Review Board IRB #STUDY00001735 & Cincinnati Children's Hospital Medical Center (CCHMC) Institutional Review Board IRB #2011-2616.

All procedures involving Human subjects were conducted in accordance with the guidelines and regulations of CSHS IRB and CCHMC IRB and adhered to the principles outlined in the Declaration of Helsinki.

Mice studies: All procedures involving mice were approved by CSHS's Institutional Animal Care and Use Committee (IACUC009979) & CCHMC's Institutional Animal Care and Use Committee.

All procedures in mice were performed in compliance with institutional guidelines of CSHS and CCHMC. ARRIVE guidelines were followed for all animal procedures.

## Data availability

The datasets and reagents generated and used in the current study are available from the corresponding authors upon request.

## Supporting information

This article contains Supporting information.

## Conflict of interest

The authors declare that they have no conflicts of interest with the contents of this article.
