# Supplementary material for: Catalytic region mimetics in Na+/H+ exchanger regulatory factor 4 suppress guanylate cyclase 2C activity to regulate enterotoxin triggered diarrhea[image]
Source: J Biol Chem. 2025 Aug 5;301(10):110559. doi: 10.1016/j.jbc.2025.110559 (PMC12510010; doi:10.1016/j.jbc.2025.110559)
Supplement: Supplementary Information [file mmc1.docx]

**Supplementary Information**

**Supplementary figure 1: NHERF4 shares significant sequence homology within GCC catalytic site**

1. Protein BLAST to show the percentage alignment of GCC with the scaffolding proteins NHERF3. NHERF3 did not show any significant sequence similarity with GCC.
2. Protein BLAST to show the percentage alignment of GCC with the scaffolding proteins NHERF4. NHERF4 contains regions especially in PDZ1 domain which exhibit significant similarity to the catalytic regions of GCC.
3. Western blot data showing the HA-immunoprecipitation of NHERF4 constructs- NHERF4-FL, -PDZ1 and -delPDZ1.Apparant differences in band intensity reflect molar concentration differences, when normalized expression levels were comparable.
